# AI-Driven Clinical Decision Support System for Automated Ventriculomegaly Classification from Fetal Brain MRI

**DOI:** 10.3390/jimaging11120444

**Published:** 2025-12-12

**Authors:** Mannam Subbarao, Simi Surendran, Seena Thomas, Hemanth Lakshman, Vinjanampati Goutham, Keshagani Goud, Suhas Udayakumaran

**Affiliations:** 1 Department of Computer Science and Engineering, Amrita School of Computing, Amrita Vishwa Vidyapeetham, Amritapuri, Clappana 690525, India; 2 Department of Neurosurgery, Amrita Institute of Medical Sciences and Research Centre, Kochi 682041, India

**Keywords:** healthcare AI, fetal brain MRI, Deep Learning, ventriculomegaly, clinical decision support

## Abstract

Fetal ventriculomegaly (VM) is a condition characterized by abnormal enlargement of the cerebral ventricles of the fetus brain that often causes developmental disorders in children. Manual segmentation and classification of ventricular structures from brain MRI scans are time-consuming and require clinical expertise. To address this challenge, we develop an automated pipeline for ventricle segmentation, ventricular width estimation, and VM severity classification using a publicly available dataset. An adaptive slice selection strategy converts 3D MRI volumes into the most informative 2D slices, which are then segmented to isolate the lateral ventricles and deep gray matter. Ventricular width is automatically estimated to assign severity levels based on clinical thresholds, generating labeled data for training a deep learning classifier. Finally, an explainability module using a large language model integrates the MRI slices, segmentation masks, and predicted severity to provide interpretable clinical reasoning. Experimental results demonstrate that the proposed decision support system delivers robust performance, achieving dice scores of 89% and 87.5% for the 2D and 3D segmentation models, respectively. Also, the classification network attains an accuracy of 86% and an F1-score of 0.84 in VM analysis.

## 1. Introduction

Ventriculomegaly (VM) is one of the most commonly seen abnormalities of the central nervous system (CNS) in prenatal screening. It is characterized by the dilation of the cerebral ventricles and is diagnosed when the atrial width of one or both lateral ventricles measures ≥10 mm on MRI scans [1,2]. Based on ventricular width, the condition is classified as mild (10–12.5 mm), moderate (12.5–15 mm), or severe (≥15 mm) [3]. Positive cases of VM have been associated with poorer neurodevelopmental outcomes [1]. A child with moderate or severe VM has a higher chance of motor and cognitive impairments due to the underdevelopment of the corpus callosum [4].

The severity classification of fetal VM is an important factor in clinical management [5]. Mild VM (10–12 mm) requires imaging to monitor progression. Moderate VM (13–15 mm) is associated with an increased risk of neurodevelopmental abnormalities and necessitates advanced neuroimaging. However, severe VM (>15 mm) carries a higher risk of adverse neurological outcomes. The extent of the disorder is largely dependent on the width of the ventricles, and a difference of only one millimeter can detect substantially different stages of the condition [1]. Hence, early finding of VM is very important to improve neurological outcomes and general prognosis [6,7,8].

For VM diagnosis, both ultrasound and MRI are widely used, but MRI provides better spatial resolution, making it effective in diagnosing VM [9]. Conventional diagnosis depends on manual segmentation and measurement of the ventricles by radiologists, a time-consuming process that is prone to inter-observer variability. Recent advances in deep learning (DL) show promise in the automated segmentation of fetal brain structures. Architectures such as U-Net, 3D U-Net, DeepLabV3+, and GAN-based models have demonstrated strong performance in fetal brain tissue segmentation and VM classification. Gopikrishna et al. employed a U-Net-based framework to segment fetal brain ventricles to measure its size [10]. Similarly, Vahedifard et al. proposed a deep learning system using a U-Net-based model for segmentation and ventricular width estimation [11]. Also, advanced approaches, such as FetalGAN, utilize generative adversarial networks to improve segmentation quality from functional fetal MRI data [12].

Despite these advancements, VM risk assessment still encounters various challenges. A significant challenge is the scarcity of labeled MRI datasets of the fetal brain for different VM severity classes, as the annotation process demands specialized clinical expertise. To reduce the manual labeling tasks, automatic labeling methods should be figured out. Also, there remains a lack of end-to-end clinical decision support systems that integrate an interactive view of the MRIs, their segmentation, measurement estimation, classification, and interpretability into a unified pipeline.

To address the challenges in analyzing fetal ventriculomegaly, we present a workflow using FeTA dataset [13]. The dataset contains high-quality 3D T2-weighted fetal brain MRI scans. Rather than relying only on standard segmentation and classification models, the pipeline is designed specifically for fetal MRI interpretation. Firstly, we select a set of informative 2D slices from each 3D volume, focusing on the region where the deep gray matter is most prominent. These slices are the input to our segmentation stage, which separates the lateral ventricles in both 2D and 3D formats. Using the segmentation masks, we calculate the ventricular width and overall volume, and then map these measurements to severity categories based on established clinical thresholds. The labeled dataset generated through this process is then used to train a lightweight deep learning model to classify Normal, Mild, Moderate, and Severe VM. To make the system easier to use in clinical settings, we add an LLM-based explanation step that provides a doctor-friendly interpretation of the results.

The uniqueness of this work lies in the design of a unified and clinically aligned pipeline for ventriculomegaly assessment. Although we rely on established models for segmentation and classification, the steps that connect these modules are new and designed specifically for fetal VM analysis. The framework introduces a targeted slice-selection strategy, together with an automated measurement scheme, for ventricular width and volume reduces the manual workload of radiologists. In addition, the automated labeling step produces a fully annotated dataset, which is one of the most challenging aspects of supervised medical image learning. The major contributions of this work are summarized as follows:

- Development of an automatic segmentation framework for fetal brain ventricles in both 2D and 3D imaging tasks.
- Design of an automated ventriculomegaly labeling method that combines ventricle width assessment with volumetric estimation as diagnostic markers.
- Implementation of a deep learning based classification model for VM severity prediction.
- Integration of a large-language-model-driven explainability module that generates human-interpretable descriptions of model outputs.
- Implementation of an interactive clinical decision support system interface that consolidates all stages of the pipeline for streamlined user interaction.

The remainder of this paper is organized as follows: Section 2 reviews the related work on fetal brain segmentation and VM classification. Section 3 describes the proposed automated pipeline for classification and clinical decision support. Section 4 presents the performance evaluation followed by concluding remarks in Section 5.

## 2. Related Works

Recently, deep learning frameworks have become popular in medical image segmentation tasks of identifying abnormalities, such as tumors and lesions. Segmentation categorizes each pixel or voxel to separate the anatomical structures or abnormalities, such as tumors, lesions, or organs, in the most accurate way [14,15,16]. Architectures based on convolutional neural networks (CNNs) [17,18,19] and transformers have utilized contextual understanding and improved segmentation accuracy [20]. Xu et al. provided a comprehensive review of deep learning methods applied to medical image segmentation [21], while several other studies have also summarized advances in this field [14,22,23,24]. Recent advances in AI-powered healthcare systems have shown great promise in addressing personalized wellness needs [25,26,27,28,29,30].

Among the numerous segmentation models developed, architectures such as UNet [31], Res-UNet [32], and DeepMedic [33] have shown good performance in various medical imaging tasks. Later, Isensee et al. introduced nnU-Net, a self-configuring framework for biomedical image segmentation that automatically adapts its preprocessing, network design, training parameters, and postprocessing strategies to new tasks without requiring manual intervention. Unlike conventional models that depend on manual tuning, nnU-Net dynamically adjusts parameters such as the number of epochs and the learning rate to achieve optimal results.

Huang et al. conducted a comparative study with three popular architectures—nnUNet, UNet, and Res-UNet—and evaluated for three medical image segmentation challenges, brain tumor, polyp, and heart ventricle segmentation [34]. The results indicated that nnUNet outperformed the other models in polyp and heart segmentation tasks and attained good results in brain tumor segmentation. Also, Kharaji et al. proposed enhancements to nnUNet by incorporating attention mechanisms [35]. In brain tumor segmentation tasks, this model showed high performance compared to standard nnUNet and DeepMedic. These results indicate that nnUnet models have a strong generalization ability across various medical imaging tasks.

Beyond nnUNet-based approaches, several methods have been proposed to improve segmentation accuracy and generalizability. For example, Ma et al. introduced MedSAM, which can segment multiple organs, tissues, and lesions [36]. Wu et al. proposed the high-order vision mamba UNet to improve global and local feature learning through state space modeling [37]. Also, Iqbal et al. developed a hybrid architecture with CNNs, vision transformers, and biconvolutional LSTMs to model both spatial and temporal dependencies, resulting in improved segmentation accuracy [19]. Junde Wu et al. proposed a Medical SAM Adapter that uses an adaptation technique incorporating domain-specific medical knowledge into the segmentation model [38]. Also, studies exploit the effectiveness of feature representations that are highly discriminative in 3D medical image segmentation. However, the challenges that are posed by a limited number of high-quality annotations, low contrast of the image, variability in the shape of the organ, and noise have negative effects on the performance. The issues are resolved by the recent methods through the means of generating pseudo-labels from pretrained models and applying confidence-based filtering, along with perturbation learning to progressively train the network.

In the field of medical image classification, a variety of CNN architectures to enhance diagnostic accuracy across diverse clinical domains. The latest DL research indicates that it is also quite accurate in classifying medical scans and segmentation masks [39]. Some of the architectures, such as ResNet [40], MobileNet [41], and EfficientNet [42] are exhibiting remarkable effectiveness. Moreover, transfer learning will allow the model to be trained with a small amount of data without the trouble of overfitting to the training data [43]. Xu et al. [40] highlighted the performance of ResNet in identifying lung tumors, breast cancer, and Alzheimer’s disease through MRI analysis. Similarly, EfficientNet employs compound scaling to jointly optimize model depth, width, and resolution, thereby attaining high accuracy with a reduced number of parameters. Zulfiqar et al. reported that a fine-tuned EfficientNetB2 model achieved remarkable results in several multi-class medical imaging tasks [42]. Also, MobileNet, which utilizes depthwise separable convolutions, provides a computationally efficient alternative. One of the notable studies came up with a framework that combines various models like CNN, ResNet50, InceptionV3, EfficientNetB0, and NASNetMobile to analyze MRI scans.

Attention mechanisms and transformer-based architectures were also used to enhance the model performance. Aftab et al. proposed a system combining inverted residual CNNs with self-attention modules [44]. Prasad et al. demonstrated deep learning integrated with cloud computing to overcome computational limitations in Alzheimer’s disease classification based on MRI [45]. Also, Ullah et al. introduced an explainable AI approach for medical image analysis to improve transparency in decision-making [46]. Emerging research, such as quantum machine learning, has also applied in medical image classification tasks to improve the performance [47,48].

The main issue in automating VM prognosis is the limited availability of labeled training data required by the supervised deep learning models. To address this issue, researchers have explored many strategies. For example, the authors in [11] utilized the correlation between deep gray matter area and ventricle size for prediction. She et al. developed a segmentation-based brain biometry system with some manual input also [49]. Gopikrishna et al. proposed a VM size estimation approach using DeepLabV3+ and U-Net architectures [10]. Another line of research [11] proposes a deep learning framework designed to automate the detection of ventriculomegaly by closely replicating the radiologist’s diagnostic workflow. Similarly, Yun et al. introduced a deep CNN-based model for fetal brain age prediction [50]. Despite recent progress, there is still a lack of publicly available, well-annotated datasets and a fully automated deep learning workflow for ventriculomegaly prognosis. To address this gap, the present work focuses on generating VM labels automatically and building a complete end-to-end pipeline capable of supporting VM assessment without manual intervention.

## 3. Automated Pipeline for Ventriculomegaly Prognosis

**Figure 1:**
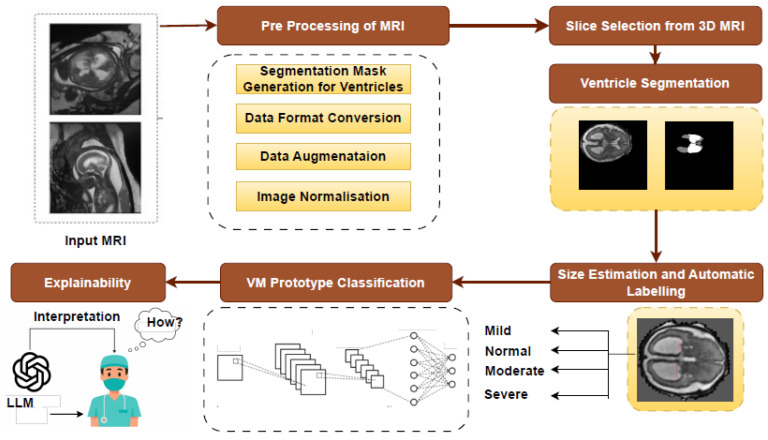
Architecture of ventriculomegaly prognosis pipeline.

The automated pipeline for ventriculomegaly prognosis is shown in Figure 1. The pipeline starts with a preprocessing step that normalizes the input data specific for VM analysis. The original dataset includes several tissue classes, but only the lateral ventricles and deep gray matter are required for our downstream tasks. Therefore, the multiclass masks are reduced to retain only the lateral ventricles and deep gray matter to ensure that the remaining stages of the pipeline operate on clinically relevant regions. This is followed by a slice selection strategy, which converts 3D MRI volumes into representative 2D slices by extracting the most informative anatomical regions. The ventricle size estimation and classification module then measures the ventricular width and categorizes each case into severity levels, such as Normal, Mild, Moderate, or Severe. A lightweight classification model is then trained on the labeled dataset. Finally, a large language model layer integrates the original MRI slice, its segmentation, and the predicted VM class to generate structured, interpretable reasoning that supports clinical understanding and trust.

The main contributions of this work lie in the design of a unified and clinically aligned pipeline for fetal MRI–based ventriculomegaly assessment. While standard architectures are used for segmentation and classification, the framework introduces several task-specific elements that are not present in existing approaches. The slice-selection strategy and automated ventricle measurement module generates a labeled dataset without the extensive manual effort typically required in fetal MRI studies.

### 3.1. Dataset Description

This study uses the publicly available FeTA 2024 Challenge dataset [51], which contains high-resolution three-dimensional T2-weighted (T2w) MRI scans of the fetal brain. It consists of 80 reconstructed volumes collected between 21 and 36 weeks of gestation. Each MRI volume includes a manually generated segmentation mask outlining seven brain tissues: cortex, white matter, external cerebrospinal fluid spaces, ventricular system, deep gray matter, cerebellum, and brainstem. Although detailed tissue-level annotations are available, this dataset does not include ventricle-level labels describing the presence or degree of VM. To generate clinically relevant annotations for subsequent classification tasks, automated labeling methods are employed as an initial step.

### 3.2. Data Preprocessing

**Figure 2:**
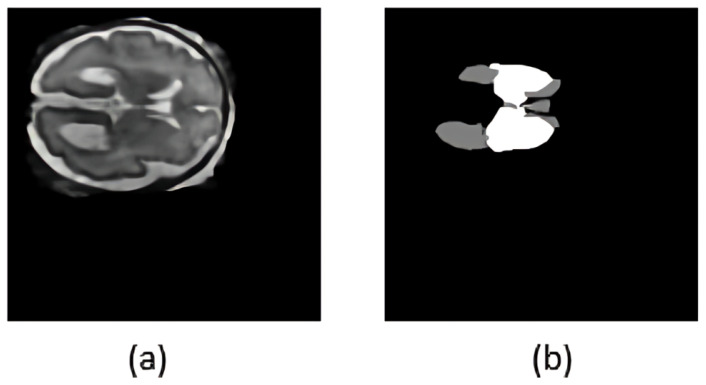
Visualization of the preprocessing step: (**a**) original T2-weighted MRI slice; (**b**) preprocessed segmentation mask, in which the ventricles are depicted in light gray and the deep gray matter in white.

The FeTA 2024 dataset provides segmentation masks defining seven distinct fetal brain tissues. Since VM evaluation depends on the lateral ventricles and deep gray matter, the segmentation labels were simplified to highlight only those regions. All other labels, such as the cortex, white matter, and cerebellum, were reassigned to background label 0. The lateral ventricles and deep gray matter were labeled as label 1 and label 2, respectively. This streamlined masks help to segment ventricles accurately. Also, an intensity normalization is carried out on each 3D MRI volume to ensure a consistent voxel intensity range across different MRIs. This step improves convergence during model training. Algorithm 1 details the preprocessing step. Figure 2 illustrates a representative T2-weighted MRI slice and its processed segmentation mask, highlighting the ventricles and deep gray matter.
**Algorithm 1:** Fetal brain MRI preprocessing
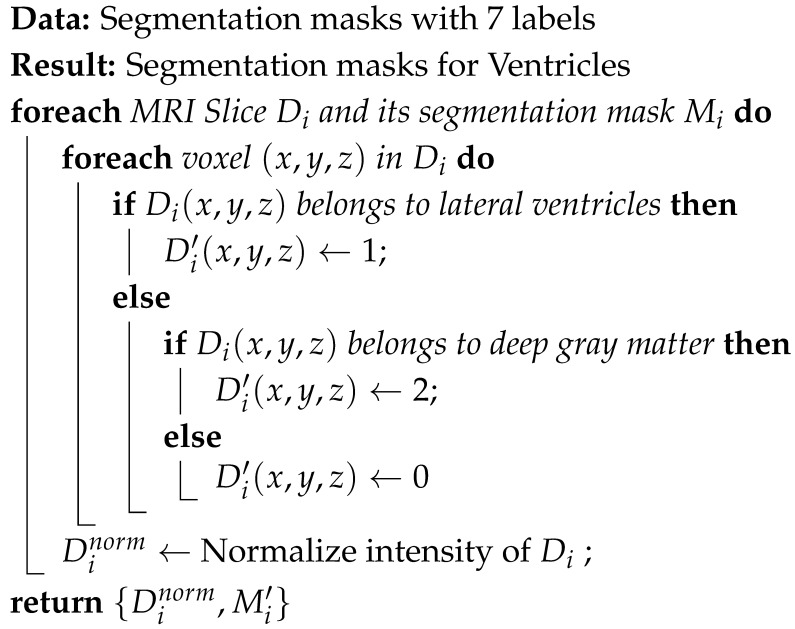



### 3.3. Slice Selection Strategy from 3D MRI Data

Although 3D MRI scans provide volumetric information required for segmentation, 2D MRI slices remain the dominant format in clinical settings because they are more computationally and storage efficient. To ensure our pipeline operates with both data types, we used a slice selection approach that converts 3D volumes into representative 2D slices. Instead of extracting all slices from the 3D scans, which would introduce redundancy and increase the likelihood of including irrelevant regions, we implemented a slice selection strategy. Algorithm 2 describes the detailed steps. This approach identifies the slice with the largest visible deep gray matter area, which corresponds to the point of maximal ventricle cross section. Once the central slice is identified, twelve slices above and twelve slices below are selected. We generated a 2D dataset of approximately 2000 anatomically meaningful slices, which are used for training and evaluating both the 2D nnUNet segmentation model and the classification network.

In fetal MRI, ventricular width (atrial diameter) is measured on an axial plane passing through the cavum septi pellucidi (CSP) and thalami. In this region, the lateral ventricles achieve their maximal cross-sectional width [52]. Our slice-selection rule follows this clinical convention by identifying the slice with maximal deep gray matter, which closely corresponds to the CSP plane, and then extracting a small neighborhood of adjacent slices to preserve local anatomical context.

To examine the robustness of this choice and understand how much surrounding anatomical information is beneficial for VM classification, we conducted a comparison using two slice ranges ±10 and ±13 slices. The results indicated that the ±10 configuration performed comparably to our baseline of 12 slices. The wider ±13 window showed a slight reduction in accuracy. This means that minor offsets produced little change in segmentation or classification accuracy. These observations support both the validity of our selection strategy and the model’s tolerance to small misalignment errors.
**Algorithm 2:** Slice selection strategy**Data**: 3D MRI volume $V_{i}$ with corresponding segmentation mask $M_{i}$**Result**: Informative 2D slices1: Extract the deep gray matter $G^{matter}$ from segmentation mask $M_{i}$2: Calculate the area of $G^{matter}$3. Identify the slice $S_{max}$ with the largest area of $G^{matter}$4: Label $S_{max}$ as the slice of maximal ventricle cross section5: Select 12 slices above and 12 slices below $S_{max}$$$Selected_{i}=\left\{S_{max-12},\ldots ,S_{max},\ldots ,S_{max+12}\right\}$$  6: Extract the slices from $V_{i}$:$$Slices_{i}=\left\{V_{i}\left(k\right)\;|\;k\in Selected_{i}\right\}$$  7: Store $Slices_{i}$ as the 2D dataset**return** $Slices_{i}$

### 3.4. Ventricle Segmentation

Ventricle segmentation is the base step in our labeling process. To utilize both 2D and 3D formats, we trained two separate segmentation models for each data type. We used nnU-Netv2, a self-configuring segmentation framework based on the U-Net architecture. nnU-Netv2 automates input normalization, resizing, data augmentation, and hyperparameter optimization. For model training, we employed a predetermined five-fold cross-validation scheme. The 3D model was trained on 70 MRI volumes, reserving 10 volumes for testing, whereas the 2D model used a larger dataset composed of 1750 images for training and 250 images for testing. Both models generated segmentation output spatially aligned with their original image inputs.

Although nnU-Netv2 provides automated configuration for most segmentation tasks, customizations are also incorporated. We manually adjusted the patch size to balance spatial coverage. The data augmentation strategy includes random brightness and contrast variations across MRI scans. For training, we employed the Adam optimizer with a learning rate of $3\times 10^{-4}$ and a cosine decay scheduler with warm restarts to promote stable convergence. Training was conducted for up to 1000 epochs, incorporating early stopping with a patience of 50 epochs to prevent overfitting. A hybrid Dice and Cross-Entropy loss was used to address the class imbalance between ventricular and non-ventricular regions. Also, a connected component filtering was added in the post-processing.

### 3.5. Adaptive Ventricle Size Estimation and Automated Labeling

**Figure 3:**
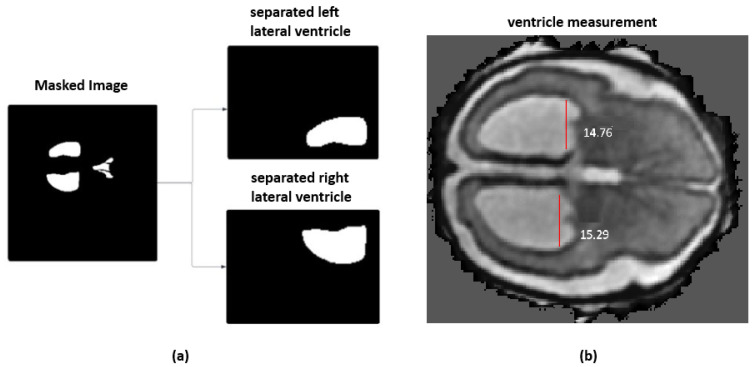
(**a**) Masked input image with adaptive ventricle splitting the proposed framework. (**b**) Automatic linear measurement of the ventricular width derived from the proposed algorithm.

On the top of ventricular size estimation algorithm, an automated labeling approach was implemented. This algorithm computes ventricular widths and annotates the MRI with severity labels. The ventricle size estimation process identifies the deep gray matter slice with the largest area. The left and right ventricles are then isolated from the segmentation mask, as shown in Figure 3a. A minimum bounding rectangle is fitted around each ventricle to calculate their widths. Unlike the methods proposed in the literature, which used a hard-coded thresholding approach to split the ventricles, the current framework introduces an adaptive partitioning mechanism. This method identifies non-null regions within the ventricle segmentation by computing row and column sums of pixel intensities and locating points of discontinuity that indicate a natural separation between ventricles. When such a separation is not clearly detectable, the algorithm divides the mask into two equal halves.

The final ventricular width is taken as the maximum of the left and right measurements and then categorized according to clinically established severity intervals, as shown in Figure 3b. When the ventricle width is measured less than 10 mm, it is labeled as normal. A width between 10 mm and 12.5 mm is considered as a mild case. For a moderate case, the width range is between 12.5 mm and 15 mm. Ventricles measuring 15 mm or greater are classified as severe. For two-dimensional MRI data, the same procedure is followed except for the slice selection stage. Algorithm 3 provides the detailed steps of the labeling process. All automatically assigned severity labels were reviewed by an experienced radiologist, assessing whether the measured widths and volumetric estimates matched expected anatomical boundaries. Approximately 90% of the generated labels were confirmed to be correct.

### 3.6. Deep Learning-Based VM Severety Classification

Deep learning classifier on 2D segmentation masks was applied to classify the different stages of VM severity. While the 2D slices had been taken from 3D MRI volumes, we narrowed down the dataset to locate the most relevant clinical slices at the level of the thalami and third ventricle areas. For each MRI, twelve slices centered around the optimal slice are identified based on the largest deep gray matter area. This resulted in a total of 852 slices, distributed across four severity categories as follows: Non-VM (420 slices), Mild VM (155 slices), Moderate VM (201 slices), and Severe VM (76 slices).

Deep learning architectures, including ResNet18, MobileNetV2, EfficientNetB0, and DenseNet121, were evaluated for VM classification. Each network was trained on 2D segmentation mask inputs, where the ventricles and deep gray matter were annotated. A lightweight, computationally efficient model, MobileNetV2 is selected as the final model without compromising accuracy. To improve convergence and stability on the small dataset, transfer learning was employed using ImageNet-pretrained weights. The dataset was divided into 70% for training and 30% for validation and testing, ensuring reliable performance evaluation.
**Algorithm 3:** Ventricle size estimation and VM class labeling
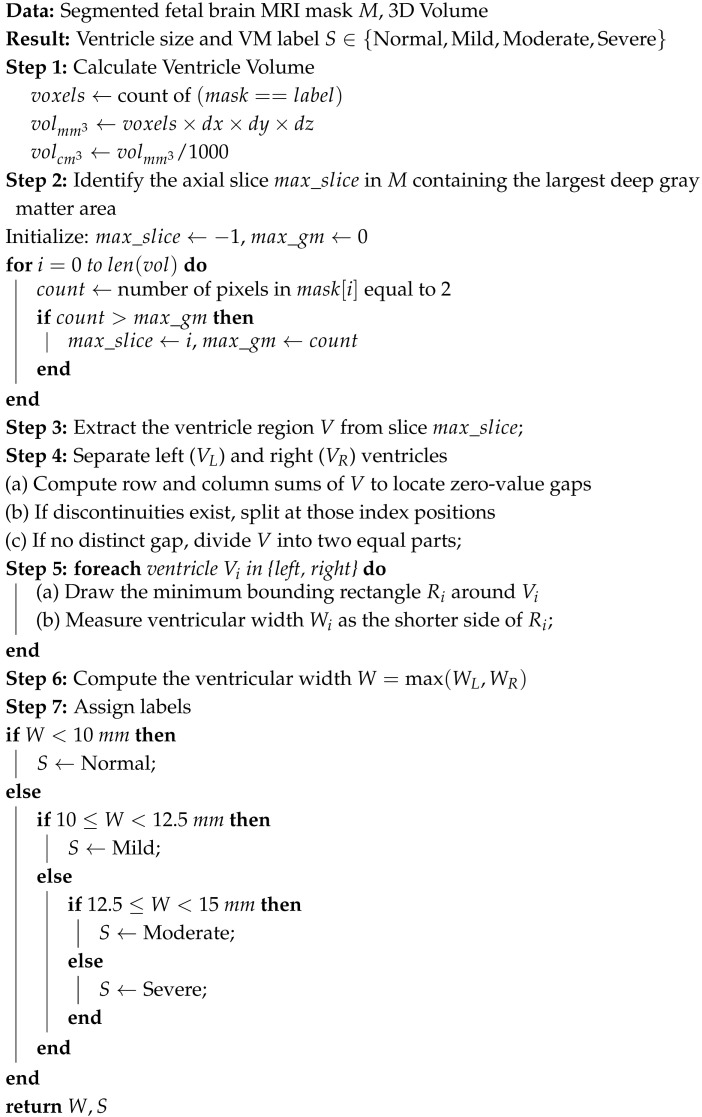



To address class imbalance and to generalize the model, we applied a set of data augmentations that included random flips, rotations, and affine transformations. In addition, class weights were incorporated into the loss function so that underrepresented categories contributed more strongly during training. We used focal loss to to give more priority for hard to classify examples and reduce the impact of easily classified samples. The Adam optimizer was used for its adaptive learning rate and efficient convergence properties. All models were trained for up to 100 epochs, with early stopping based on validation loss to prevent overfitting and ensure optimal performance.

### 3.7. LLM-Based Explanation Generation

To improve the clinical usability of the proposed pipeline, an LLM-based module was integrated which generates natural language explanations for the classification results. This will assist clinicians in interpreting automated predictions. The LLM module uses the original 2D MRI slice, its corresponding segmented slice, and the predicted VM severity class (Normal, Mild, Moderate, or Severe) as its input. These elements are formatted into a structured prompt for model’s reasoning. The resulting text is given to the Google Gemini API that provides a clinically relevant explanation. Prompts are formatted in a template-driven structure with class-specific cues. For instance: *Slice: [Image]; Severity: Moderate VM; Width: 13.2 mm; Volume: 3.6 cm^3^*, the explanation is *“The lateral ventricles exceed the 12.5 mm threshold, indicating moderate ventriculomegaly. Volume supports abnormal fluid accumulation”*. The automatic reporting and question-answering capability provides additional support to radiologists and clinicians, making the interpretation of analysis results.

## 4. Performance Evaluation

### 4.1. Implementation Details

For the evaluation of the pipeline, we conducted all experiments on a Windows 10 workstation equipped with an Intel Core i7 processor operating at 3.0 GHz, 16 GB of RAM, and an NVIDIA RTX 2070 GPU with 8 GB of VRAM. Deep learning components were implemented in Python 3.9 using PyTorch 1.10. GPU acceleration was enabled through an NVIDIA CUDA-supported graphics card with 4 GB of VRAM. Version mismatches of CUDA–PyTorch were diagnosed using the nvidia-smi utility. The entire software stack with the model weights and the interface components requires about 2 GB of free disk space. Segmentation methods were based on the nnUNetv2 framework, and for classification, different deep learning models were employed. To make the processes of training, evaluation, and inference reproducible, Jupyter notebooks, PyQt-based graphical user interface modules, and shell automation scripts were used.

In order to support a steady convergence and reduce the risk of overfitting, a cosine decay scheduler with warm restarts was employed. The training was limited to 1000 epochs; however, early stopping was implemented after 50 consecutive epochs so as to terminate unnecessary training. A composite loss function combining Dice loss and cross-entropy was adopted to balance region-level overlap accuracy. The segmentation pipeline generated binary masks delineating the ventricles and deep gray matter. Post-processing procedures were subsequently applied. These include including morphological closing to smooth anatomical boundaries and the removal of small spurious regions. An interactive user interface was integrated to run the entire pipeline. Users could select either 2D or 3D analysis mode, loading MRI data in PNG or NIfTI format. LLM-based explanations were generated through the Google Gemini API. For users relying on external API-based explainability features, incorrect keys could be updated through the GUI or manually adjusted in the configuration file.

We evaluated our proposed approach with 2D and 3D baseline models. For the segmentation task, both the 2D and 3D versions of nnU-Net were included, as this framework is widely regarded for its strong performance across many biomedical datasets. For the ventriculomegaly classification component, we trained a set of standard 2D convolutional models such as ResNet18, MobileNetV2, EfficientNet-B0, and DenseNet121. All of these baselines were run under the same conditions, with identical preprocessing, subject-level data splits, augmentation settings, training schedules, and evaluation metrics. To quantify model variability and ensure statistically reliable comparisons, each experiment was repeated with multiple random seeds. For all metrics, we report the mean and standard deviation across these runs. Across all repeated runs, our method demonstrated low variance, indicating stable behavior with respect to different random initializations. All metrics are reported as mean ± standard deviation over *N* independent runs.

For the segmentation task, we ensured leakage-safe and reproducible dataset handling prior to training the nnU-Netv2 models. All 2D slices and 3D volumes were grouped at the subject level. A fixed random seed was used for all experiments. Following this, both 2D and 3D nnU-Netv2 configurations were trained using its self-configuring features, which automatically determine patch size, model depth, batch size, and learning rate. All models were trained using the Adam optimizer with a learning rate of $3\times 10^{-4}$, cosine decay with warm restarts, and a combined Dice + Cross-Entropy loss for 1000 epochs with early stopping. This ensures consistent and reproducible training behavior across runs. For classification, the dataset was randomly divided into training, validation, and test sets using a fixed seed. The model training was performed using ImageNet-pretrained weights with the Adam optimizer and focal loss with class-balanced weighting. The class distribution consisted of 420 Non-VM, 155 Mild VM, 201 Moderate VM, and 76 Severe VM samples; to address this imbalance, weighted sampling and targeted augmentation were applied consistently across all folds. All experiments were repeated using the same seeds to ensure reproducibility.

To support reproducibility, we outline the full preprocessing and configuration steps used throughout the pipeline. All MRI scans were first resampled to a uniform voxel spacing and oriented to a consistent anatomical reference. Intensity values were z-score normalized on a per-volume basis after clipping outlier values beyond the 0.5th and 99.5th percentiles. For 3D volumes, our slice-selection module identified the slice with maximal deep-gray-matter representation, and extracted ±12 neighboring slices to create a standardized 25-slice stack. For segmentation, we used nnU-Netv2 with its self-configuring 2D and 3D pipelines. The models were trained using Dice + Cross-Entropy loss, Adam optimizer, and cosine learning-rate scheduling. For classification, all 2D backbones received fixed-size inputs of 224 × 224 pixels. Preprocessing included resizing, center cropping, and identical augmentation policies across models. We detail learning rates, batch sizes, class weights, and all hyperparameters used for each classifier. Subject-level splitting was enforced to prevent leakage, and random seeds used for each run are documented.

### 4.2. Segmentation Performance

**Figure 4:**
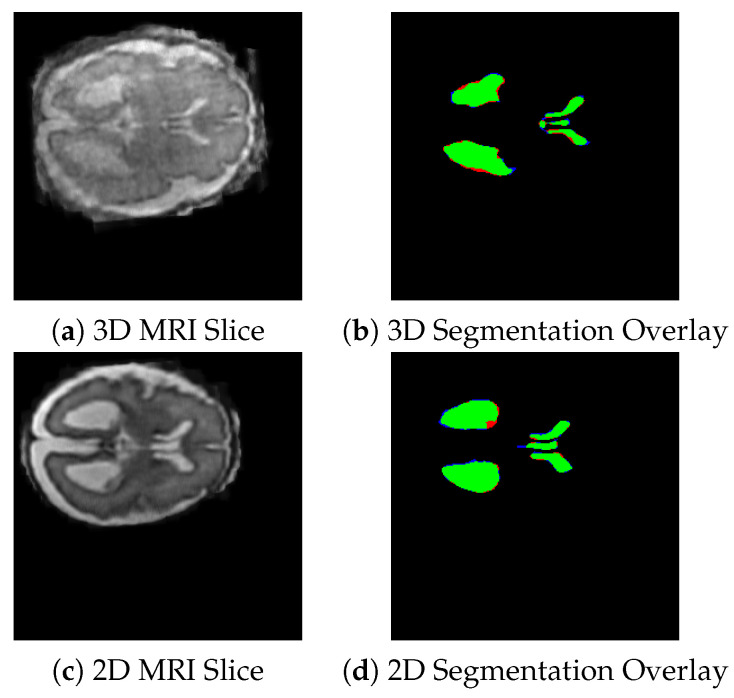
Visualization of MRI inputs and corresponding segmentation outputs. (**a**) 3D MRI slice showing anatomical structure. (**b**) 3D segmentation overlay with model predicted regions within the 3D volume. (**c**) 2D MRI slice extracted from the volumetric scan. (**d**) 2D segmentation overlay with predicted boundaries on the 2D slice.

We evaluated the performance of both 2D and 3D nnUNet models on ventricle and deep gray matter segmentation. The 2D model is evaluated for ventricular segmentation, while the 3D model segments the ventricles and deep gray matter. Dice score and Intersection over Union (IoU) were used as evaluation metrics. Figure 4 shows the visual representation of the segmentation masks generated by the 3D nnUNet (top row) and 2D nnUNet (bottom row) models on the selected fetal brain MRI slices.

The summary of segmentation performance of 3D and 2D nnUNet models are presented in Table 1. The Dice score for ventricles of the 2D nnUNet was 0.889. For 3D nnUNet, the Dice score for ventricles is 0.875 and for deep gray matter is 0.790, but the average Dice score is 0.834 across regions. The associated IoU scores are 0.78 for ventricles and 0.75 for deep gray matter. These results indicate that both models are effective in segmenting the ventricles, with 2D nnUNet achieving a slightly better performance. We have also tested the convergence behavior of the two models. The 3D nnU-Net converged faster (405 epochs) than the 2D model (966 epochs). This indicates that the 3D network benefits from more contextual information. However, the 3D model learns faster, and it may require additional optimization strategies to improve the segmentation performance compared to 2D model.

As shown in Figure 4, the green areas are the regions that were correctly identified (true positives), the red areas are those have been missed (false negatives), and the blue areas are those that have been over-segmented (false positives).

### 4.3. Classification Performance

In the dataset, VM category labels were assigned using the automated ventricle size estimation module of the pipeline. To ensure clinical reliability, the labeled cases were then independently reviewed by radiologists. Overall, 89% of the labeled cases were confirmed as accurate by the radiologists. This reviewed dataset was subsequently used for model training and evaluation.

**Figure 5:**
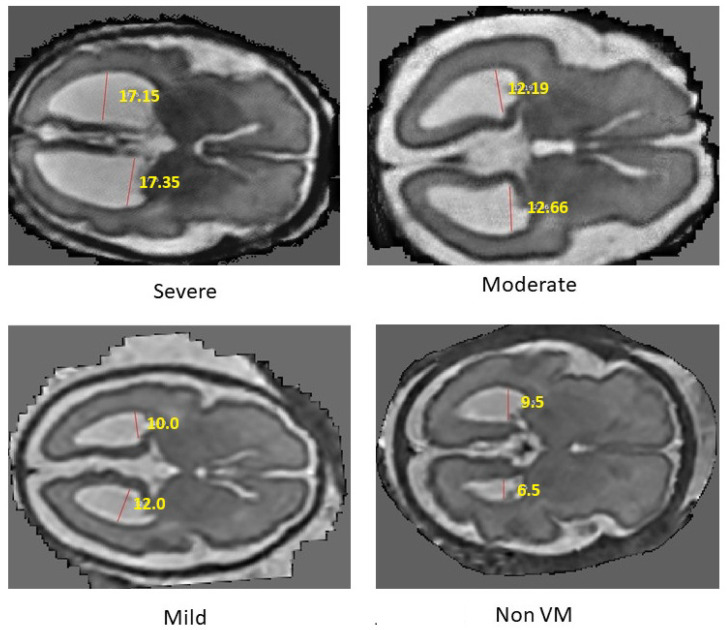
VM severity annotation and classification.

**Figure 6:**
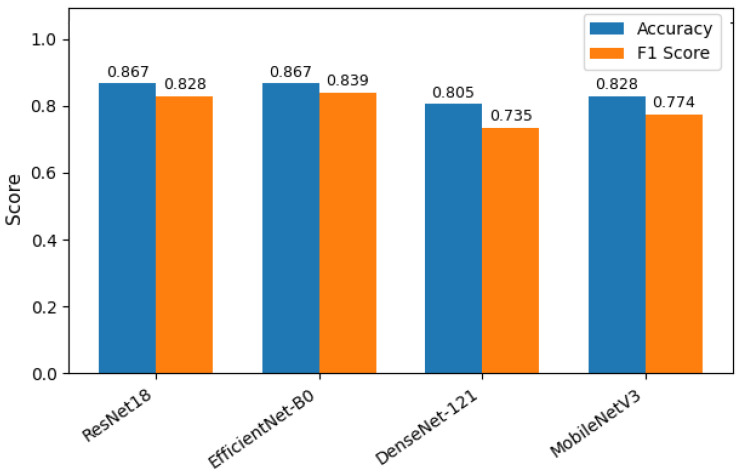
Comparison of macro F1-scores across models.

Four deep learning models—ResNet18, MobileNetV2, EfficientNetB0, and DenseNet121—were evaluated for classifying the severity of ventriculomegaly. The models were classified into four categories, Non-VM, Mild VM, Moderate VM, and Severe VM, as shown in Figure 5. Performance was assessed using accuracy, precision, recall, and F1-score metrics, with particular emphasis on class-wise and macro-average values due to the imbalanced class distribution. Table 2 shows the accuracy and macro F1 score of these models. EfficientNetB0 demonstrated the highest overall performance, achieving an accuracy of 86.7% and a macro F1-score of 0.839. ResNet18 exhibited comparable accuracy but slightly lower F1 performance, suggesting less balanced classification across severity levels. MobileNetV2 and DenseNet121 achieved moderately good metrics. The comparative results, illustrated in Figure 6, show EfficientNetB0 as the most generalizable model for VM severity classification.

To better understand the per-class performance, Table 3 presents precision, recall, and F1-score for each class. Non-VM and Severe VM were consistently classified with high accuracy across all models, while Mild and Moderate VM classes were more prone to misclassification. EfficientNetB0 demonstrates the most balanced performance across all classes, particularly for the Severe and Non-VM categories, achieving high precision (0.846–0.939) and strong recall (0.984–1.000). Its consistently high F1-scores indicate good generalization across severity levels.

**Figure 7:**
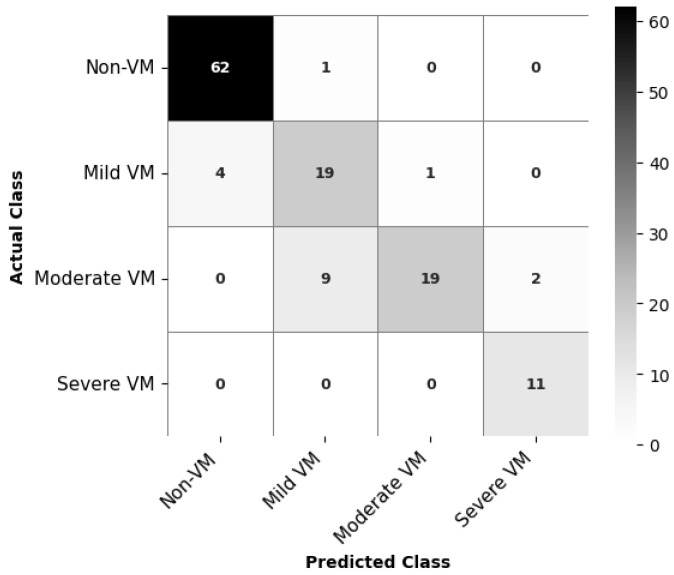
Confusion matrix for EfficientNetB0 for the class-wise prediction results for VM severity classification.

The confusion matrix for EfficientNetB0, as depicted in Figure 7, indicates class-wise prediction performance of the classification model for VM severity. The model achieves high accuracy for Non-VM and Severe VM classes, correctly classifying 62 out of 63 Non-VM cases and all 11 Severe VM cases. For Mild VM, 19 out of 23 cases are correctly identified, but there are some misclassifications, with 4 cases predicted as Non-VM. The most challenging category appears to be Moderate VM: while 19 cases are correctly classified, the model confuses 9 Moderate VM cases as Mild VM and 2 as Severe VM. This is understandable given the slight anatomical differences.

**Figure 8:**
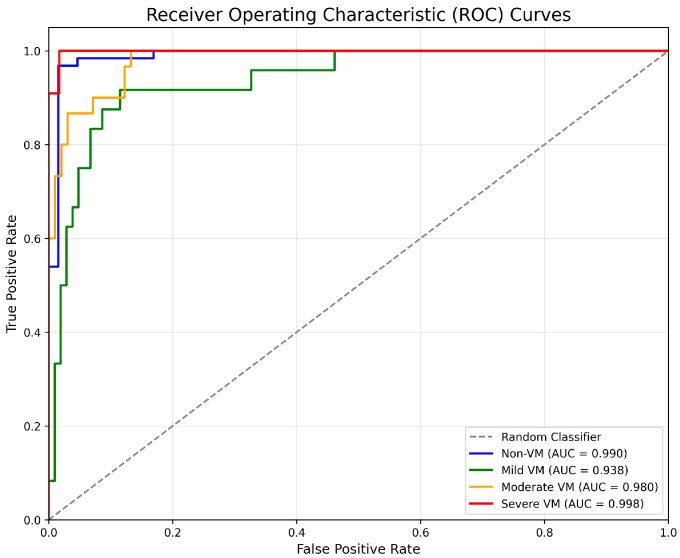
EfficientNetB0 ROC curves.

The effectiveness of EfficientNetB0 is also supported by the ROC curves in Figure 8. All classes achieve very high AUC values, with Non-VM and Severe VM with values AUC = 0.990 and 0.998, respectively. Moderate VM also achieves good performance of AUC = 0.980, while Mild VM with a slightly lower AUC = 0.938.

Furthermore, we developed a decision support system to assist clinicians in the analysis of fetal brain MRI scans for VM assessment. The graphical user interface provides an interactive environment where radiologists can visualize the original T2-weighted MRI slice and its segmentation for the comparison of anatomical structures, such as the lateral ventricles and deep gray matter. In the user interface, users could select either 2D or 3D analysis mode, load MRI data in PNG or NIfTI format, and initiate processing through the Begin Analysis option. The interface displayed results across two dedicated panels: an Imaging Results view showing the original scan with the segmentation overlay, and a Clinical Analysis view presenting VM severity classification along with the LLM-generated explanation.

**Figure 9:**
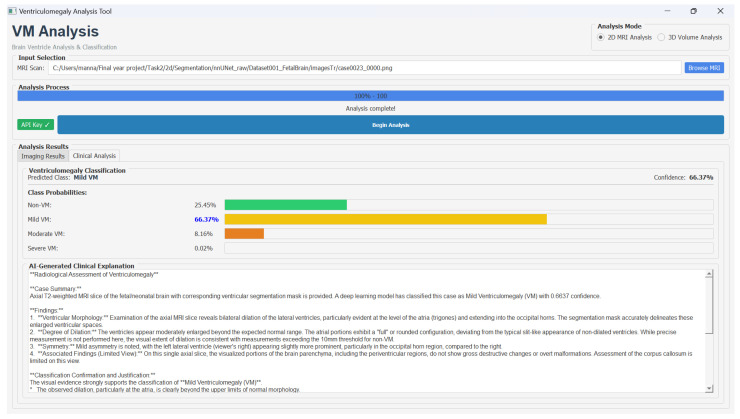
Graphical user interface of the ventriculomegaly analysis tool for fetal brain MRI: Original T2-weighted MRI slice and its processed segmentation mask, class-wise probability distribution, and an auto-generated clinical explanation produced by Gemini API.

The integrated classification associated with class-wise probability distribution provides a clear view of the VM severity levels. The clinically interpretable explanations using the Google Gemini API helps the clinicians to trust the model results. Hence, this decision support system provides data-driven diagnostic assistance by combining multi-modal visualization, deep learning-based analysis, and explainable AI reasoning. Figure 9 shows the VM analysis tool and the classification results.

To evaluate the behavior of the LLM module in a clinically meaningful way, we conducted a small qualitative review with three radiologists. The LLM produced brief, structured explanations based solely on the pipeline’s outputs (ventricle width, volume, and predicted class). An example output was “The lateral ventricles measure 13.1 mm at their widest point, which places this case in the moderate VM range. No significant asymmetry is seen. A routine postnatal MRI may be considered”.

Clinicians consistently reported that the explanations were clear and easy to read, and that they could be helpful when preparing patient-facing summaries. They also noted that the module behaved appropriately under the guardrail instructions we provided; i.e., it remained within the supplied measurements and avoided speculative statements. The main caveat identified was reduced clarity in borderline cases, where the model’s output lies near the Mild/Moderate threshold.

These qualitative findings complement our quantitative analysis, showing that nnU-Net segmentation paired with EfficientNet classification forms a reliable backbone for VM detection, while the LLM enhances interpretability and user acceptance. At the same time, the feedback highlights an important next step: explanation generation should be better aligned with model uncertainty, so that borderline predictions are communicated with appropriate caution. We have now added this point to the Section 4.4 as a limitation and a direction for future refinement.

### 4.4. Discussion

The proposed method demonstrates that it can perform well in identifying via segmentation and classification. The 2D nnUNet of the segmentation yielded better Dice (0.889) and IoU (0.806) results than the 3D nnUNet (Dice: 0.875, IoU: 0.790), which indicates that the 2D model can localize and delineate ventricles in single slices more accurately. The higher IoU of the 2D model thus confirms that it can capture shape and boundary overlap with the ground truth—a very important aspect in medical segmentation. The reason for the 2D model outperforming the 3D model could be that the 2D model only focused on the segmentation of ventricles, while the 3D model was performing the dual task of ventricle as well as deep gray matter segmentation. The 3D model may have had trouble deep gray matter accurately (Dice: 0.790, IoU: 0.75), therefore resulting in the overall performance being lower.

In the case of classification, EfficientNetB0 was the most effective model out of the four tested in terms of the trade-off between accuracy (86.7%) and macro F1-score (0.839). What drove the model to essentially learn distinct imaging features in these two categories Non-VM (F1: 0.961) and Severe VM (F1: 0.917) is its excellent performance in them. The architectural efficiency of EfficientNetB0 that achieves network depth and width optimization through compound scaling is the most plausible explanation for why it works well for medical imaging tasks when data is scarce.

One of the major issues for the classification of medical images was the identification of the Mild and Moderate VM categories. These categories are, by their nature, very hard to separate, as they are based on slight anatomical differences and have overlapping visual features. The confusion matrices and class-wise F1 scores show the ambiguity of the situation. Although the classification model can distinguish between VM and Non-VM, it cannot differentiate between Mild and Moderate. Also, in segmentation, the deep gray matter presented more challenges for the delineation, particularly in the 3D model, due to their lower contrast, smaller size, and less distinct boundaries. These issues may have had a greater impact on the IoU, as it penalizes partial overlaps more severely than Dice.

## 5. Conclusions

This paper presented an automated AI-driven clinical decision support system for fetal brain MRI analysis, for the assessment of ventriculomegaly. The proposed pipeline integrates preprocessing to generate ventricle and deep gray matter segmentation masks, automatic slice selection, and ventricle size estimation and severity labeling. A deep learning-based classifier was employed to categorize disease severity into four classes that demonstrates strong performances. In addition, the system incorporates a large-language-model-based explanation module that generates structured, interpretable clinical insights to support radiologists in diagnostic reasoning. This decision support system serves as a platform, combining anatomical visualization, automated classification, and explainable AI outputs to reduce manual effort and enhance diagnostic efficiency. Future work will focus on expanding the dataset for higher generalizability and refining the explainability component. The findings of this work apply only to the FeTA dataset used for training and evaluation. Although FeTA offers good-quality fetal MRI scans, it includes relatively few subjects, lacks multi-center representation, and provides limited metadata. These factors restrict our ability to judge how well the method would generalize across scanners or clinical settings. Assessing broader applicability will require testing on larger, multi-center datasets with richer annotations in future studies.

## Figures and Tables

**Table 1:** Ventricle segmentation performance of 2D and 3D nnUNet models.

Model	Region	Dice Score	IoU Score
3D nnUNet	Ventricles	0.875	0.78
	Deep Gray Matter	0.790	0.75
	Average	0.834	0.76
2D nnUNet	Ventricles	0.889	0.81

**Table 2:** Summary of accuracy and macro F1-score of VM classification models.

Model	Accuracy	Macro F1-Score
ResNet18	0.867	0.828
ResNet50	0.887	0.832
MobileNetV2	0.828	0.774
EfficientNetB0	0.867	0.839
DenseNet121	0.805	0.735

**Table 3:** Per-class performance metrics for classification models.

Model	Precision				Recall				F1-Score			
	Non-VM	Mild	Moderate	Severe	Non-VM	Mild	Moderate	Severe	Non-VM	Mild	Moderate	Severe
ResNet18	0.925	0.824	0.781	0.833	0.984	0.583	0.833	0.909	0.954	0.683	0.806	0.870
ResNet50	0.932	0.831	0.820	0.844	0.986	0.620	0.840	0.915	0.955	0.699	0.825	0.879
MobileNetV2	0.897	0.708	1	0.579	0.968	0.708	0.567	1	0.931	0.708	0.724	0.734
EfficientNetB0	0.939	0.655	0.950	0.846	0.984	0.792	0.633	1	0.961	0.717	0.759	0.917
DenseNet121	0.873	0.786	0.792	0.579	0.984	0.598	0.633	1	0.925	0.579	0.703	0.734

## Data Availability

The data presented in this study are openly available in FETA2024 at https://fetachallenge.github.io/pages/Data_download.html (accessed on 12 September 2024).
